# Spermatogonial stem cell markers and distribution in different regions of the testis of adult Nile tilapia (*Oreochromis niloticus*)

**DOI:** 10.1590/1984-3143-AR2025-0162

**Published:** 2026-07-27

**Authors:** Sunny Okechukwu Abarikwu, Samyra Maria dos Santos Nassif Lacerda, Guilherme Mattos Jardim Costa, Luiz Renato de França

**Affiliations:** 1 Department of Biochemistry, University of Port Harcourt, Choba, Nigeria; 2 Laboratório de Biologia Celular, Departamento de Morfologia, Universidade Federal de Minas Gerais – UFMG, Belo Horizonte, MG, Brasil

**Keywords:** Tilapia (*O. niloticus*), spermatogonial stem cells (SSCs), SSCs niche, SSCs markers, testicular development

## Abstract

Highly conserved vertebrate molecular markers, such as Gfra1, Pou5f3, Notch1, Plzf, and Nanos2, represent important candidates for evaluating spermatogonial stem cells (SSCs) physiology and preferential location in the fish testis. In tilapia, germ cell cysts are distributed within seminiferous tubules oriented along the dorso–ventral axis, while the adult testis continues to grow along both the longitudinal (cranio–caudal) and dorso–ventral axes. Herein we investigated the spatial distribution of undifferentiated spermatogonia (A_und_) expressing established SSC-associated markers in the testes of sexually mature Nile tilapia. Topographical analysis along the longitudinal axis showed that A_und_ were predominantly located in regions of the seminiferous tubules adjacent to the interstitial compartment, with approximately 53% of A_und_ in the caudal region and 18% in the cranial region of the testis. Accordingly, most A_und_ positive for Gfra1, Plzf, and Nanos2 (>70%; p<0.05) were preferentially detected in the caudal region. Analysis along the dorso–ventral axis showed that A_und_ positive for Plzf, Gfrα1, Nanos2, Nanos1, Notch1, and Notch3 were more frequently observed in areas closer to the tunica albuginea compared with regions adjacent to the efferent ducts. Together, these findings demonstrate pronounced regional differences in the distribution of A_und_ along both the cranio–caudal and dorso–ventral axes of the Nile tilapia testis, highlighting a spatially organized germinal architecture that may be relevant for the maintenance of the germinal epithelium and post-pubertal testicular growth. To our knowledge, this is the first study to systematically describe the spatial distribution of A_und_-associated markers in a teleost species.

## Introduction

In Nile tilapia, the testis is elongated along the cranio–caudal axis, while the seminiferous tubules are organized dorso–ventrally, forming a characteristic architectural pattern in this species (Alvarenga and França, 2009; Pfennig et al., 2012; Palladino et al., 2023; Setthawong et al., 2024). Furthermore, full spermatogenesis is established when body and testis size are still small in Nile tilapia, as they grow continually well beyond puberty (Manosroi et al., 2004; Kefi et al., 2012).

Although studies from our research group have suggested a dorso-ventral difference in the distribution of undifferentiated spermatogonia in the Nile tilapia testis (Lacerda et al., 2013), the mechanisms underlying testicular growth in sexually mature fish remain incompletely understood. In this context, the possibility that spatially organized regions at the cranial and/or caudal ends of the gonad contribute to germ cell distribution, analogous to organizing regions described during embryonic development that involve distinct gene expression cascades (Dequéant and Pourquié, 2008; Thönnes et al., 2022), should also be considered.

Fish A_und_ spermatogonia, also considered spermatogonial stem cells (SSCs), are well-characterized isolated germ cells that show a prominent nucleolus and low nuclear heterochromatin (Schulz et al., 2010; Lacerda et al., 2014; Diao et al., 2022). This morphological profile closely resembles that described in mammals, in which the SSC population is primarily harbored within type A single spermatogonia (A_s_), supporting the notion that key cytomorphological features of SSCs are evolutionarily conserved across vertebrates (de Rooij and Russell, 2000; Grisanti et al., 2009; Yoshida, 2012). Only a limited number of specific germ cell markers have been used to characterize SSCs in fish testes (Bosseboeuf et al., 2013; Lacerda et al., 2013; Xie et al., 2020). In mammals, the glial cell-derived neurotrophic factor (GDNF) has been identified as the main factor for SSC self-renewal and maintenance. In the adult Nile tilapia testis, the expression profile of GDNF family receptor alpha 1 (GFRa1) is specifically found in single A_und_, particularly in cells located in distal seminiferous tubule regions adjacent to the tunica albuginea (Lacerda et al., 2013; Rajachandran et al., 2023; Liu et al., 2024).

Other candidate markers of germline stem cells include members of the Nanos gene family. Nanos genes encode evolutionarily conserved zinc-finger RNA-binding proteins that play important roles for germline stem cell function (Draper et al., 2007; Chen et al., 2025). GFRa1^+^/Nanos2^+^ spermatogonial populations are known to largely correspond to A_s_ and A paired (A_pr_) spermatogonia in mammals, which are potential stem cells in the undisturbed testis (Bosseboeuf et al., 2013; Bellaiche et al., 2014; Xie et al., 2020). In this regard, GDNF signaling is essential to maintain Nanos2 expression in murine undifferentiated spermatogonia and the overexpression of Nanos2 can alleviate the stem cell loss phenotype caused by the depletion of the Gfra1 gene (Sada et al., 2012; Doretto et al., 2022). Our previous observations with Nile tilapia testis also suggested that Nanos2 expression is found in isolated A_und_ and in small clones of germ cells considered differentiated spermatogonia (A_diff_), and is not found in more advanced germ cells such as type B spermatogonia, spermatocytes, spermatids and spermatozoa (Lacerda et al., 2013).

The transcriptional factors promyelocytic leukemia zinc finger protein (PLZF) and Pou domain class 5 homeobox 1 (POU5F1/OCT4, termed Pou5f3 in teleosts) are also important molecular markers of the A_und_ population, which includes SSCs of mammals and some fish species (Takehashi et al., 2012; Bosseboeuf et al., 2013). Pou5F1/Oct4 is a member of the class V POU-domain transcription factor family and its homologues, now named Pou5f3, have been identified and described in teleost fishes. In testis, Pou5F1/Oct4 is an important marker of pluripotency for the spermatogonial lineage and is involved in the maintenance of stem cell fate (Maekawa et al., 2024; Ikeda et al., 2025). In most species investigated to date, from medaka to mammals, expression of Pou5f family members was reported in primordial germ cells during development and in A_und_ spermatogonia in adults (Encinas et al., 2012; Froschauer et al., 2013; Zhong et al., 2021). In the adult zebrafish testis, Plzf was found in the nucleus of both type A and type B spermatogonia (Leal et al., 2009) and its mRNA expression was detected in A_und_ and A_diff_ spermatogonia as well as in meiotic cells and young spermatids of dogfish testes (Bosseboeuf et al., 2013). Our previous observations in the testis of adult Nile tilapia revealed that Pou5f3 expression is predominantly localized in A_und_ situated in the seminiferous tubule regions adjacent to the tunica albuginea (Lacerda et al., 2013), a pattern also reported in the dogfish (*Scyliorhinus canicula*) testis (Bosseboeuf et al., 2013). In addition, higher Plzf and Pou5f3 mRNA levels were detected in Jundia (*Rhamdia quelen*) testicular cell fractions enriched by A_und_ (Ozaki et al., 2011; Mohapatra and Barman, 2014; Bellaiche et al., 2014; Shang et al., 2015; Lacerda et al., 2019), and other fish species including zebrafish and sablefish (Voronina and Pshennikova, 2016; Hayman et al., 2021; Qian et al., 2022).

Notch-1 (neurogenic locus notch homolog protein 1-like) is specifically involved in germ cell differentiation in humans (Hayashi et al., 2004; Sambe et al., 2023), whereas in rodents its expression starts before birth in gonocytes and increases as the germ cells proliferate and differentiate into type A and B spermatogonia, showing a peak in spermatocytes (Kostereva and Hofmann, 2008; Khanehzad et al., 2021). In our preliminary studies on Nile tilapia testis, Notch1 receptor expression was detected in A_und_ located in the seminiferous tubule regions adjacent to the tunica albuginea (Lacerda et al., 2013), suggesting that activated Notch1 may play a role in cell fate determination, as well as in the maintenance and differentiation of spermatogonial cells.

Therefore, to gain insights regarding the cellular events that promote testis growth in sexually mature fish, the present study aimed to further characterize the Nile tilapia testis parenchyma. We were particularly interested in investigating the distribution and preferential location of A_und_ along the dorso-ventral and longitudinal (cranio-caudal) axes of the Nile tilapia testis, using several well-established vertebrate SSCs markers, such as Gfra1, Pou5f3, Plzf, Notch1, Nanos1 and Nanos2.

## Methods

### Experimental animals, sampling and tissue preparation

**Figure 1:**
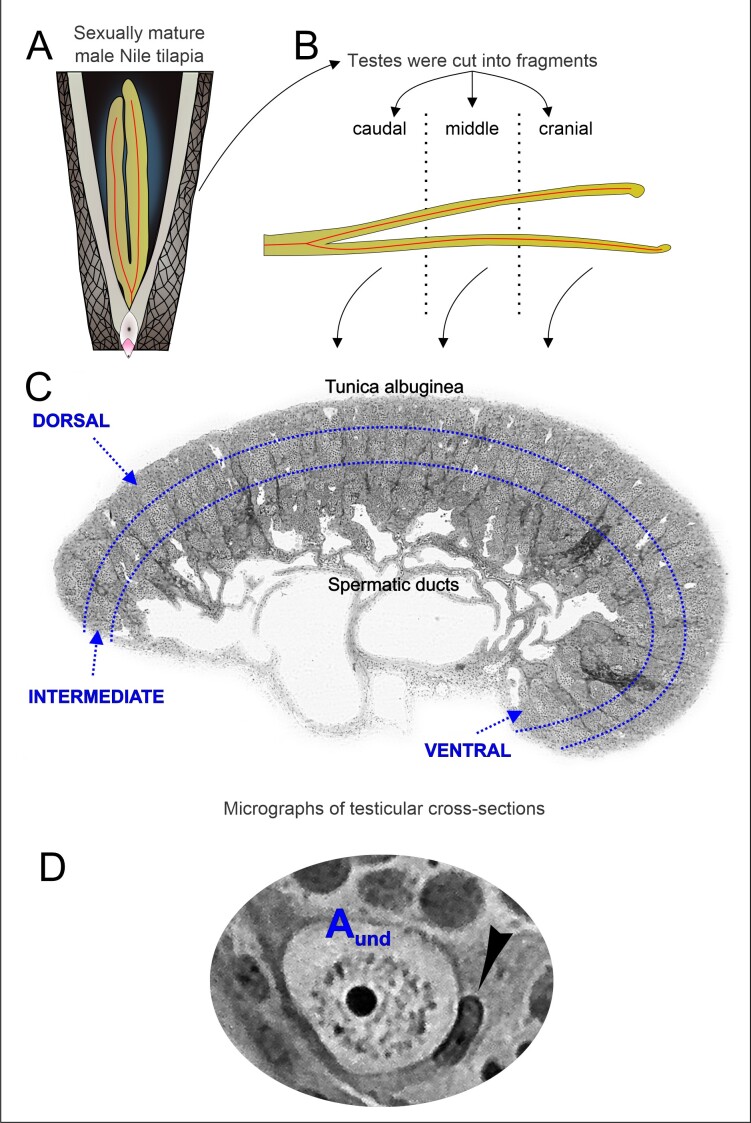
Schematic diagram of experimental design. The testes from young sexually mature Nile tilapia (A) were removed and subsequently cut perpendicularly into fragments (cranial, middle, and caudal) along the longitudinal axis (B). These fragments were then fixed and embedded, and micrographs of the histological cross-sections were analyzed in the different regions of the testicular parenchyma (C), i.e., dorsal (close to the tunica albuginea), intermediate and ventral (close to the spermatic duct region). Micrographs were digitally standardized and divided into three equal areas along the defined anatomical axis, ensuring proportional and reproducible regional segmentation. (D) Undifferentiated type A spermatogonia (A_und_) in Nile tilapia testis, characterized as large single cells with a round nucleus and a prominent nucleolus, surrounded by Sertoli cells (arrowhead).

Ten young sexually mature male Nile tilapia (*Oreochromis niloticus)*, approximately five months of age, were obtained from the commercial aquaculture station 3D Aqua Ltda (Morada Nova de Minas, MG) located in the Southeast region of Brazil. The fish were maintained under 12L:12D light–dark cycle for 4 weeks, at approximately 25 °C, and were fed *ad libitum* with commercial pellets every day. All experimental procedures were reviewed and approved by the Ethics Committee on Animal Use (CETEA) of the Federal University of Minas Gerais, Brazil, under approval number 89/2012, and were conducted in accordance with institutional and international guidelines for the ethical treatment of animals. Body weight was measured after euthanasia by Quinaldine anesthesia (90% GC; Sigma-Aldrich, St. Louis, MO, U.S.A.). The paired testes were then removed and weighed. After that, the testes were then sectioned by hand into small fragments using a razor blade, perpendicularly to the longitudinal axis, corresponding to the cranial, middle, and caudal regions (Figures 11B). The caudal region was sampled near the caudally located urogenital papilla, the middle region was taken halfway between the caudal and cranial testis tips and the cranial region was sampled near the cranial tip of the testis. The testis was divided into three equal parts, with the caudal region defined as the portion closest to the urogenital papilla, representing the distal 33.3% of the testis length. After histological processing, testicular cross-section micrographs were first oriented using anatomical landmarks to define the dorso–ventral axis. Digital micrographs were then standardized and divided along this axis into three equal proportional areas, designated as dorsal (adjacent to the tunica albuginea), intermediate, and ventral regions (adjacent to the spermatic duct). The intermediate region was defined as the central third between the dorsal and ventral regions. The same proportional segmentation criterion was applied consistently to all micrographs, ensuring objective regional assessment (Figure 1C). The tissue fragments were collected from all ten fish evaluated after regional dissection. Thereafter, the fish were then divided into two experimental groups where five fish were used for quantitative morphometric analysis and five for immunohistochemical identification of A_und_. Accordingly, tissue fragments from the morphometric group were fixed in buffered 4% glutaraldehyde, whereas those from the immunohistochemical group were fixed in 4% paraformaldehyde. Samples were embedded in glycol methacrylate (Historesin® - Leica Instruments, Heidelberg, Germany) for morphological analysis, whereas those used for immunohistochemistry were embedded in Paraplast (Sigma-Aldrich, St. Louis, MO, USA).

### Topographical distribution of type A undifferentiated (A_und_) spermatogonia in the seminiferous tubules

A_und_ were identified according to the morphological features described previously (Schulz et al., 2010; Lacerda et al., 2014; Siqueira-Silva et al., 2021; Xie et al., 2020). The topographical distribution of A_und_ was recorded by examining if A_und_ were adjacent to the interstitial compartment (with or without blood vessels), or in contact with one or more tubules (intertubule). The numbers of A_und_ were counted in the caudal, middle and cranial regions, and the position of 200 A_und_ per animal was evaluated and expressed as a percentage of the total number analyzed. To assess the positional association of these cells with testicular compartments, the tubular perimeters adjacent to the interstitium or intertubule were measured, using ImageJ software (downloaded from NIH, 2025), and the values were expressed as percentage of the total tubular perimeter (n= 50 tubules/fish).

### Immunostaining analyses

To characterize the A_und_ in the cranio-caudal and dorso-ventral axes of the Nile tilapia testes, we performed immunostaining using the immunoperoxidase method and serial sections 5 μm thick were analyzed by light microscopy (BX-60 Olympus). Tissue sections were immunostained using protocols specifically developed for each antigen and with antibody dilution previously tested. Following dewaxing and rehydration, antigen retrieval was performed in 0.1 M citrate buffer (pH 6.0), after boiling for 10 min in a microwave oven. Endogenous peroxidase was quenched for 30 min with 0.6% H_2_O_2_ (Sigma-Aldrich, St. Louis, MO, U.S.A.) in TBS. Non-specific binding was blocked with 10% normal goat, rabbit, or horse serum (Sigma-Aldrich) in 1% BSA in TBS. The tissue sections were stained for the following quite well established spermatogonial markers: Gfra1; Nanos2; Nanos1; Notch1; Notch3; Plzf; and Pou5f3 (see Table 1 for additional details of primary antibodies). Because the primary antibodies were originally raised against antigens from other vertebrate species, immunolabeling specificity in Nile tilapia was assessed based on: (i) prior optimization of antibody dilution and staining conditions for each antigen; (ii) conserved and cell type-restricted immunoreactivity patterns across biological replicates; and (iii) agreement of the observed labeling with the expected distribution of morphologically identified A_und_ and with previous reports in Nile tilapia and other teleosts. The slides were then incubated overnight at 4 °C. Biotinylated anti-rabbit IgG (Abcam, ab6720, 1:200), anti-goat IgG (Abcam, ab6740, 1:100), and anti-mouse IgG (Vectastain®, Vector Laboratories, 1:200) antibodies were applied and incubated for 60 min, at room temperature. Detection of the signal was performed by incubating the sections in streptavidin-HRP for 15 min, followed by the reaction with peroxidase substrate diaminobenzidine and counterstaining with hematoxylin (Merck), also at room temperature. Following dehydration, tissue sections were mounted and analyzed. Negative controls were performed in each immunohistochemical run by omission of the primary antibody, which resulted in the absence of specific staining. External positive-control tissues were not included due to the limited availability of standardized Nile tilapia reference tissues for all evaluated targets. The number of A_und_ was recorded in the caudal, middle, and cranial regions, and a total of 200 A_und_ per animal was evaluated across these regions. Their position was then expressed as the percentage of the total number of A_und_ analyzed.

**Table 1:** List of antibodies and manufacturers used in the present study.

Epitope	Manufacturer	Catalogue Number	Dilution	Secondary Antibody (IgG)
GFR*α*1	Abcam	ab84106	1:500	Goat anti rabbit
Nanos2	Abcam	ab169436	1:40	Horse anti-mouse
Nanos1	Abcam	ab65203	1: 50	Goat anti-rabbit
Notch1	Santa Cruz	sc-3299	1:100	Rabbit anti-goat
Notch3	Santa Cruz	sc-32347	1:100	Rabbit anti-goat
PLZF	Santa Cruz	sc-11146	1:100	Rabbit anti-goat
Pou5f1/Oct4	Abcam	ab18976	1:100	Goat anti-rabbit

### Statistical analyses

After testing for normality using the Shapiro–Wilk test, with all data sets found to be normally distributed around the mean, significant differences between two-parameter analyses were performed using Studentʼs t-test. Comparisons of more than two groups were performed with one-way ANOVA, followed by Student-Newman-Keuls test. This approach allowed us to control for type I error while comparing multiple groups. All statistical analyses were performed using GraphPad Prism v6.0 (GraphPad Software, San Diego, CA, USA), and the obtained data were expressed as mean ± SD and statistical significance was set at p < 0.05.

## Results

### Biometric data and topographic distribution of A_und_ in the seminiferous tubules

**Figure 2:**
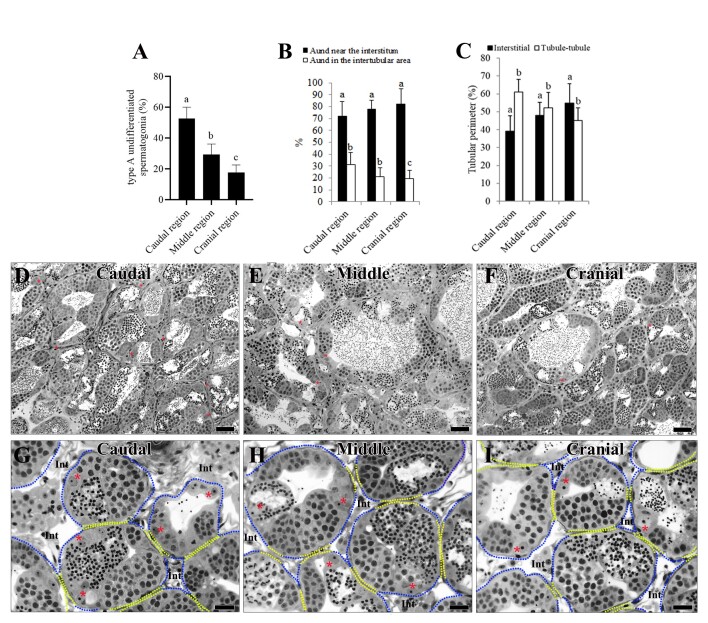
Topographic distribution of A_und_ in the Nile tilapia testes. (A) Quantification of A_und_ in the caudal, middle, and cranial regions of the testicular parenchyma, where a significantly higher concentration of A_und_ is observed in the caudal region; (B) Note that in these regions most of the A_und_ are preferentially located near the interstitium; (C) Percentage of the tubular perimeter of the regions contacting the interstitium or tubule-tubule contact. Observe that, except for the cranial region, the tubule-tubule contact is significantly higher in the two other evaluated regions. Data represent means ± SD (n=5). Bars with different letters are significantly different among regions (p<0.05). Cross-sections of the seminiferous tubules of the Nile-tilapia testes (D-I) showing most of the A_und_ (asterisks) distributed near the interstitium (Int). Caudal end (D and G), middle region (E and H), cranial end (F and I). Tubule-tubule contact areas are outlined in yellow, and tubule-interstitial contact areas are marked in blue (G-I). Staining: Toluidine blue. Scale bar = 500 µm in D-F; 30 µm in G-I.

Final body weight and mean testis weight for the Nile tilapia investigated in the present study were 320 ± 4 g and 1.35 ± 0.35 g, respectively, providing a gonadosomatic index (testis mass divided by body weight) of 1.04 ± 0.28%. Morphologically, the A_und_ have large nucleus with a prominent nucleolus and occur as single cells along the seminiferous tubules, where they are surrounded by Sertoli cells (Figure 1D). The topographical distribution of A_und_ in the different regions of the tilapia testes showed that 53 ± 10% of these cells were found in the caudal region, 29 ± 8% in the middle region, and 18 ± 6% in the cranial region (Figure 2A), and most of these cells (~70-80%) were found close to the interstitial compartment (Figures 2B, DI) even though this compartment represented around half (~40% to ~55%) of the total tubular perimeter (Figure 2C).

### Immunostaining of A_und_ markers in the longitudinal and dorso-ventral axes of tilapia testes

**Figure 3:**
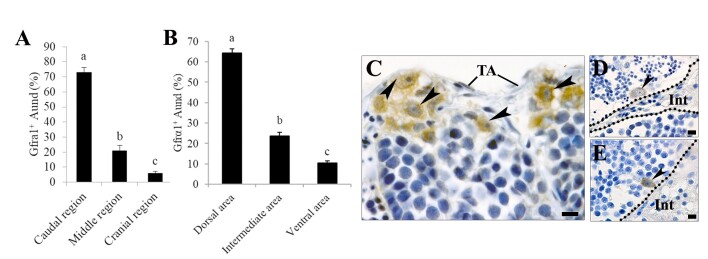
Quantitative analysis of Gfra1-positive A_und_ in the caudal, middle, and cranial regions along the longitudinal (A) and the dorso-ventral (B) axes of the Nile tilapia testis. Observe that, in comparison to the other investigated regions/areas, there is a significantly higher predominance of positive cells in the dorsal area and caudal region (p<0.05). Data represent means ± SD (n=5). Bars with different letters are significantly different among regions (p<0.05). Transversal testis sections of the Nile tilapia testis (C) illustrating a higher density of Gfra1-positive spermatogonial cells (arrowheads) at the distal region of the seminiferous tubules, near to tunica albuginea (TA). (D-E) In other seminiferous tubules areas, Gfra1^+^ cells were frequently found as single spermatogonial cells (arrowhead) near the interstitial compartment (Int). The interstitium is delimited by spotted lines. Staining: Immunoperoxidase staining with hematoxylin counterstaining. Scale bar = 10 µm.

**Figure 4:**
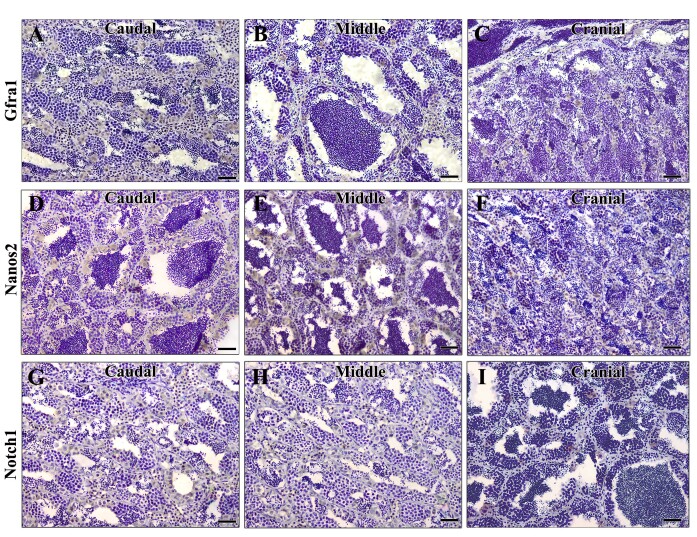
Representative images of Nile tilapia testicular parenchyma immunolabeled with anti-Gfra1 (A-C), anti-Nanos2 (D-F), and anti-Notch1 (G-I) in the caudal, middle, and cranial regions of the testes. Staining: Immunoperoxidase staining with hematoxylin counterstaining. Scale bar = 60 µm.

Using morphological characterization and Gfra1 immunolabeling, the quantitative evaluation of spermatogonial cell distribution in the caudal, middle and cranial regions of the testes showed that 73 ± 2%, 21 ± 3.6% and 6 ± 2.1% were Gfra1^+^ A_und_, respectively (p<0.05; Figure 3A). In the dorso-ventral axis, these cells were mainly located close to the tunica albuginea and were less frequently seen close to the ductal region (64 ± 1.8%, 23 ± 1.6%, 10%± 0.8 respectively (p<0.05) (Figures 33C). In all regions (caudal, middle and cranial) Gfra1^+^ cells were found more frequently as single spermatogonial cells near to the interstitial compartment (p<0.05; Figures 33E). As expected, most of these cells were morphologically characterized as A_und_spermatogonia (Figures 3CE). More advanced germ cells such as type B spermatogonia, spermatocytes, spermatids and spermatozoa, as well as somatic cells, including Sertoli cells and Leydig cells, did not show Gfra1 immunoreactivity. Finally, regarding Gfra1 marker and considering the three different regions evaluated along the longitudinal axis, stronger immunostaining was observed in the caudal region, than in the cranial region (Figures 4AC).

**Figure 5:**
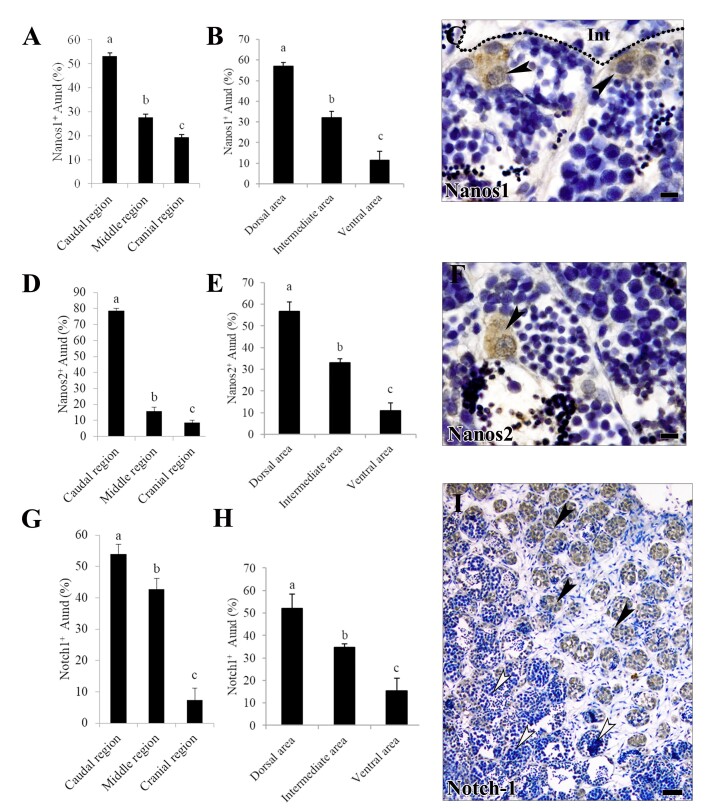
Quantitative analysis of positive Nanos1, Nanos2, and Notch1 A_und_ in the caudal, middle and cranial regions along the longitudinal (A, D, G) and the dorso-ventral (B, E, H) axes of the Nile tilapia testis. Observe that, in relation to the other investigated regions/areas, there is a significant predominance of positive cells in the dorsal and caudal region/area (p<0.05). Data represent means ± SD (n=5). Bars with different letters are significantly different among regions (p<0.05). Immunohistochemical localization of Nanos1 (C), Nanos2 (F), and Notch1 (I) protein in the sexually mature Nile tilapia. Immunoreactivity is frequently found in early type A spermatogonia (arrowheads in C and E). Note that, in specific areas of the seminiferous tubules located adjacent or close to the tunica albuginea, i.e. the seminiferous tubules blind ending (I, black arrowheads), it is possible to observe a higher concentration of Notch1^+^ spermatogonia, when compared to the other areas along the dorso-ventral axis (I, white arrowheads). The interstitium (Int, C) is delimited by spotted lines. Staining: Immunoperoxidase staining with hematoxylin counterstaining. Scale bar = 10 µm in C and F; 500 µm in I.

Similarly, in comparison to the cranial tip, in the longitudinal axis the immunoreactivity of anti-Nanos1, anti-Nanos2, anti-Notch1, anti-Notch3, anti-Plzf, and anti-Pou5f3 were found more frequently in the caudal region of the testes, as well as closer to the tunica albuginea in the dorsal-ventral axis. Therefore, using both morphological and Nanos1 immunolabeling approaches along both axes (Figures 5AC), the quantitative evaluation of Nanos1^+^ A_und_ distribution in the caudal, middle and cranial regions were, respectively, 53 ± 2%, 28 ± 1% and 19 ± 1% (p<0.05; Figure 5A). Also, in comparison to the ventral side, Nanos1^+^ undifferentiated spermatogonial cells were more frequently observed on the dorsal area (Figures 55C). Accordingly, Nanos2 immunolabeling in the caudal, middle and cranial region of the testis showed that 79 ± 2%, 16 ± 3%, and 9 ± 2% of A_und_ spermatogonia were respectively Nanos2^+^ (p<0.05; Figures 55F; Figures 4DF). Following the expected trend, in comparison to the ventral side Nanos2^+^ A_und_ were more frequently found in the dorsal region of the testis (Figure 5E). Regarding Notch1, immunolabeling in the caudal, middle and cranial regions of the testis showed that 54 ± 3%, 43 ± 5%, and 7 ± 4% of A_und_ spermatogonia were, respectively, Notch1^+^ cells (p<0.05; Figures 5GI and Figures 4GI).

**Figure 6:**
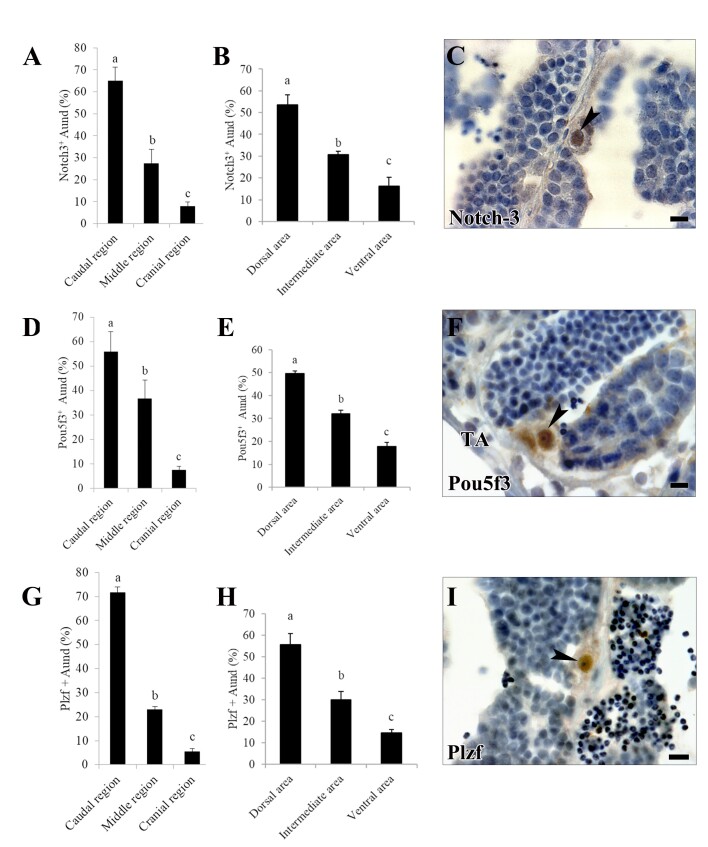
Quantitative analysis of positive Notch3 (A), Pou5f3 (D), and Plzf (G) A_und_ in the caudal, middle and cranial regions along the longitudinal (A, D, G) and the dorso-ventral (B, E, H) axes of the Nile tilapia testis. Note that, in comparison to the other investigated regions/areas, there is a significant predominance of positive cells in the dorsal and caudal region/area (p<0.05). Data represent means ± SD (n=5). Bars with different letters are significantly different among regions (p<0.05). Immunohistochemical localization of Notch3 (C), Pou5f3 (F), and Plzf (I) protein in the adult Nile tilapia testis. Immunoreactivity is frequently found in A_und_ (arrowhead) located near to the tunica albuginea (TA). Staining: Immunoperoxidase staining with hematoxylin counterstaining. Scale bar = 10 µm C, F, and I.

**Figure 7:**
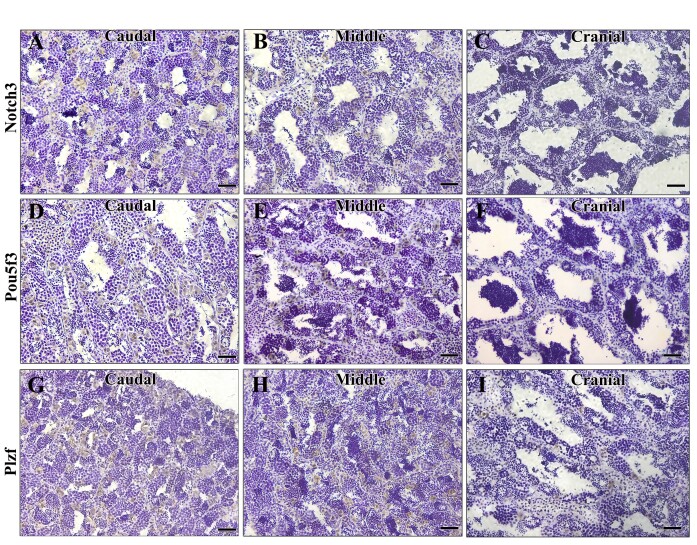
Representative images of Nile tilapia testicular parenchyma immunolabeled with anti-Notch3 (A–C), anti-Pou5f3 (D–F), and anti-Plzf (G–I) in the caudal, middle, and cranial regions of the testes. Staining: Immunoperoxidase staining with hematoxylin counterstaining. Scale bar = 60 µm.

Figure 6 shows the immunolabeling for Notch3, Pou5f3 and Plzf. Consistent with the pattern described above, in this Figure it can be observed that 65 ± 3%, 27 ± 11%, and 8 ± 3% of Notch3^+^ A_und_ spermatogonia were present, respectively, in the caudal, middle and cranial region of the testis, and a similar trend was found for the dorsal area in comparison to the ventral area (p<0.05; Figures 6AC and Figures 7AC). Furthermore, it should be mentioned that both the Notch1^+^ and Notch3^+^ spermatogonia presented a preferential location towards the dorsal side, near the tunica albuginea, in comparison to the ventral side near the ductal region (Figures 5 H and 6B). Pou5f3 immunolabeling in the caudal, middle and cranial region of the testis showed that 56 ± 8%, 37 ± 8%, and 8 ± 1% of A_und_ spermatogonia were, respectively, Pou5f3^+^ (p<0.05; Figures 6DF and Figures 7DF). Plzf immuno-positive A_und_ in the caudal, middle and cranial region of the testis were 72 ± 2%, 23 ± 1%, and 5 ± 1%, respectively (p<0.05; Figures 6GI and Figures 7GI). Finally, based on both qualitative and quantitative analyses, Pou5f3^+^ and Plzf^+^ spermatogonia were also mainly located on the dorsal side near the tunica albuginea and less frequently found on the ventral side near the ductal region of the testis (p<0.05; Figures 66F and Figures 6HI).

## Discussion

In our previous work with Nile tilapia, we demonstrated that established mammalian SSC markers, including Gfra1 and Nanos2, reliably identify A_und_ in fish testis (Lacerda et al., 2013). In the present study, we substantially extend these observations by showing that additional and evolutionarily conserved vertebrate markers, such as Pou5f3, Notch1, Plzf, Nanos1, and Notch3, are also highly expressed in A_und_. To our knowledge, this is the first demonstration of Plzf-, Notch3-, and Nanos1-positive A_und_ in sexually mature Nile tilapia.

Notably, the expression levels of these markers are significantly higher in the caudal region than in the cranial tip of the testis, highlighting the caudal region as a major site of A_und_ accumulation and SSC marker enrichment. Further, our analysis along the dorso-ventral axis demonstrates that A_und_ are preferentially located near the tunica albuginea, suggesting a spatially polarized distribution of SSCs to maintain spermatogenic activity across both longitudinal and dorso-ventral axes, which may be relevant for the maintenance and post-pubertal remodeling of the germinal epithelium.

Expression of molecular markers in the testes can shift when SSCs lose “stemness,” often as they leave the stem cell niche toward more differentiation-prone microenvironments in response to physiological demands (DeFalco et al., 2015; Voigt et al., 2023; Cai et al., 2025). In Nile tilapia, our observations support the existence of heterogeneous A_und_ populations distributed across different testicular regions, with areas of higher SSC density likely reflecting zones of preferential niche occupancy rather than directional cell movements.

In medaka (*Oryzias latipes*) and zebrafish (*Danio rerio*), germ cells functioning as “reserve” versus “active” stem cells have been described (Nakamura et al., 2010; Nobrega et al., 2010). Therefore, along the longitudinal axis of the Nile tilapia testis, a heterogeneous population of A_und_ likely exists, distributed across region-specific microenvironments and potentially occupying distinct functional states, according to local and systemic demands (Glauche et al., 2009; Ahmed et al., 2023). Based on the spatial enrichment patterns observed here, one may hypothesize that cranial and caudal compartments differentially support A_und_ populations with more “active” or more “reserve-like” properties, as proposed in other fish models (Xie et al., 2020). Nevertheless, such a distinction cannot be established from the present descriptive and immunohistochemical data alone and should be considered a testable hypothesis requiring direct functional validation.

We also observed that somatic cell distributions mirror those of A_und_. Sertoli cell proliferation is higher when associated with A_und_, suggesting a polarized and region-specific organization along the dorso–ventral axis (Schulz et al., 2005). Literature reports indicate that Sertoli cells with stem‐like properties are preferentially located at the blind ends of seminiferous tubules near the tunica albuginea, associated mostly with undifferentiated spermatogonia, with proliferative activity decreasing toward ductal (ventral) regions where germ cells undergo differentiation (Schulz et al., 2005; Batlouni et al., 2009; Xie et al., 2020). Conversely, Leydig cell precursors clusters are located near the spermatic ducts, where differentiated Sertoli cells and more advanced cysts predominate (Schulz et al., 2005; Xie et al., 2020). In these regions, close to the seminiferous tubules blind-ending, Sertoli cells contacting A_und_ express anti-Müllerian hormone, possibly contributing to the modulation of Leydig cell proliferation. This spatial architecture is consistent with our data, highlighting that high numbers of A_und_ coincide with regions of higher Sertoli mitotic activity, consistent with a model in which Sertoli cells potentially provide new niches prior to colonization by undifferentiated spermatogonia (França et al., 2015).

Extending our morphological observations, we found that A_und_ immunopositive for Gfra1, Nanos1, Nanos2, Plzf, Notch1, Notch3, and Pou5f3 are preferentially localized sub-albugineally and are significantly enriched in the caudal portion of the testis. Most of these expressing cells are positioned adjacent to the interstitial compartment: i.e., over 70% of A_und_ express Gfra1, Nanos2, or Plzf; more than 60% express Notch3; over 50% express Pou5f3 or Nanos1. This polarized distribution suggests not only different states or phenotypes of A_und_ (e.g. more “stem-like” vs more “primed for differentiation”) but also that the interstitial compartment (for instance, Leydig cells and vasculature) is a key component of the SSC niche that helps regulate these phenotypes (Nobrega et al., 2010; Lacerda et al., 2013; Xie et al., 2020).

Previous studies in Nile tilapia testes have shown that Gfra1 labelling is restricted to A_und (_Lacerda et al., 2013; Liu et al., 2024). In other teleost, such as rainbow trout and turbot, both GDNF and GFRa1 are expressed in type A spermatogonia, suggesting the existence of potential autocrine/paracrine loops (Nakajima et al., 2014; Duan et al., 2024). Thus, our data support and deepen these findings, showing regional enrichment of Gfra1^+^ A_und_ in the caudal regions of the testis. Given the established role of GFRa1/RET in mediating SSC self-renewal via GDNF signaling in mammals and particularly evident in fish (Naughton et al., 2006; Gautier et al., 2014; Liu et al., 2024), this spatial pattern likely reflects or reinforces a functional niche in the caudal region of the Nile tilapia testis.

Although its molecular localization is fairly well documented here, the functional characterization of Plzf in teleost testes remains incompletely understood. In zebrafish, Plzf localizes in the nucleus of both type A and early type B spermatogonia (Ozaki et al., 2011; Doretto et al., 2022). High Plzf transcript levels have been reported in SSC‐enriched fractions of A_und_ from *R. quelen* testes (Lacerda et al., 2019) and in early spermatogonia of channel catfish (*Ictalurus punctatus*) and blue catfish (*I. furcatus*) (Shang et al., 2015). Also, in spawning trout testes Plzf is immunodetected in type A spermatogonia (Bellaiche et al., 2014). Functional evidence, e.g., in *Labeo rohita*, shows PLZF acting as a transcriptional repressor critical for maintaining SSC undifferentiated state (Mohapatra and Barman, 2014). In this study, clusters of Plzf^+^ spermatogonia in the blind ends of seminiferous tubules in caudal regions suggest local foci of active spermatogonial self-renewal, reinforcing a central role for PLZF in long-term germline maintenance in fish.

Nanos2 expression in adult fish gonads has been documented in zebrafish and medaka spermatogonia and oogonia, and also in both A_und_ and A_diff_ of Nile tilapia (Aoki et al., 2009; Lacerda et al., 2013). In transplantation assays in trout, Nanos2 is observed in a subset of spermatogonia with high stemness potential, making it a useful marker to investigate SSC fate decisions between self-renewal vs. differentiation (Bellaiche et al., 2014). In Nile tilapia herein investigated, ~80% of Nanos2^+^ spermatogonia are observed in caudal testis ends, in comparison with ~9% at the cranial tips. Such a marked polarization suggests that Nanos2^+^ A_und_ serve as key intrinsic regulators of the undifferentiated state, preventing premature differentiation (Liu et al., 2014) acting in concert with extrinsic niche cues, which remain to be fully elucidated.

In addition to Nanos2, Gfra1, and Plzf labeling, Pou5f3, Nanos1, Notch1, and Notch3 are predominantly concentrated in the sub-albugineal and caudal regions. As previously established, Pou5f3/Oct4 is a central regulator of stemness (Encinas et al., 2012; Bosseboeuf et al., 2013), whereas the Notch signaling pathway contributes to the balance between self-renewal and differentiation, via interactions with Sertoli and possibly Leydig cells (Garcia et al., 2013; Xie et al., 2020). Our findings in the present investigation align with observations in medaka, zebrafish, rainbow trout, Nile tilapia, Japanese flounder, large yellow croaker, and grouper, where Pou5f3 and/or Nanog expression is restricted to early germ cells (Sánchez-Sánchez et al., 2010; Wang et al., 2011; Gao et al., 2013, 2017; Lacerda et al., 2019; Zhong et al., 2021). Thus, we confirm Pou5f3 as a conserved SSC marker in Nile tilapia and demonstrate its region-specific expression as part of the structural organization of SSC niches. In addition, Nanos1 expression in this compartment may point to a complementary role to Nanos2 in safeguarding germ cell identity and regulating the transition between self-renewal and differentiation (Blanes-García et al., 2024). Nevertheless, further functional studies would be valuable to clarify the specific roles of Nanos1, Notch1, and Notch3 in maintaining SSC identity, and coordinating interactions within the testicular microenvironment in teleosts.

## Conclusion

Using morphological and immunolabeling approaches, we demonstrated that A_und_ are preferentially located adjacent to the interstitial compartment rather than at tubule–tubule interfaces, with their highest abundance in the caudal region of the Nile tilapia testis. Together, these findings indicate that the expanding ends of the testis rely to a large extent on self-renewing spermatogonia in the caudal region, likely sustained by signals from the interstitial compartment. By providing relevant morphofunctional insight into the tilapia gonad, we provide the first robust evidence of a polarized and region-specific spatial distribution of A_und_ along both the longitudinal (caudal > middle > cranial) and dorso–ventral (sub-albugineal > intermediate > ductal) axes of the Nile tilapia testis. Importantly, the spatial organization of putative SSCs revealed here not only advances our understanding of germline biology in teleosts but also establishes a practical anatomical framework for reproductive biotechnologies. The identification of caudal and sub-albugineal SSC-enriched domains may enable targeted tissue sampling, more efficient SSC isolation and enrichment, and improved outcomes in spermatogonial transplantation. In addition, these findings provide a rational basis for the optimization of in vitro SSC culture and cryopreservation protocols, directly supporting the establishment of germplasm banks and long-term genetic resource preservation. By precisely defining region-specific SSC localization, this study also strengthens the feasibility of surrogate broodstock strategies, genetic improvement programs, and SSC-mediated gene editing approaches. Ultimately, fish species exhibiting polarized testicular organization and spatially restricted SSC niches, such as Nile tilapia, emerge as robust model systems for germline manipulation and the development of next-generation biotechnologies aimed at sustainable aquaculture and conservation of aquatic biodiversity.

## Data Availability

Research data are available upon request.
